# Integrated pharmaceutical and behavioral strategies for long-term control of RSV transmission: An impulsive SEIRS-type modeling study

**DOI:** 10.3934/publichealth.2026046

**Published:** 2026-08-17

**Authors:** Juan Pablo Gutiérrez-Jara, María Teresa Muñoz-Quezada, Fernando Córdova-Lepe

**Affiliations:** 1 Centro de Investigación de Estudios Avanzados del Maule (CIEAM), Vicerrectoría de Investigación y Postgrado, Universidad Católica del Maule, Talca, Chile; 2 Escuela de Salud Pública, Facultad de Medicina, Universidad de Chile, Santiago, Chile; 3 Facultad de Ciencias Básicas, Universidad Católica del Maule, Talca, Chile

**Keywords:** respiratory syncytial virus, pharmacological interventions, non-pharmacological intervention, mathematical model, public health

## Abstract

Human respiratory syncytial virus (RSV) remains a leading cause of pediatric acute respiratory infections worldwide, with epidemiological patterns shifting in the aftermath of the COVID-19 pandemic. This study evaluates the independent and combined effects of pharmacological interventions and a targeted communication campaign aimed at modifying population behavior to reduce infection rates, applying an impulsive SEIRS differential equation model. The findings underscore the importance of integrated prevention strategies that combine pharmaceutical and non-pharmaceutical measures to achieve sustained control of RSV transmission and mitigate the risk of seasonal hospital surges.

## 1. Introduction

Human respiratory syncytial virus (RSV) is a primary cause of acute respiratory infections (ARIs) in infants and children under two years of age [1]. Classified within the *Pneumoviridae* family, RSV is responsible for a significant proportion of pediatric hospitalizations for bronchiolitis and pneumonia, especially in low- and middle-income countries [2]. This virus is divided into two main antigenic subtypes: A and B, which co-circulate seasonally. Studies have reported that both subtypes can cause severe infections. Although previous research suggested that subtype A might be associated with greater clinical severity [3], more recent evidence indicates that there are no conclusive differences in overall seriousness between the two [4]. However, specific variations in clinical symptoms, immune response, and viral load have been observed by age and sex [5]. These characteristics underscore the importance of continuous epidemiological monitoring and sustained preventive strategies, particularly given the potential for mutations that could alter their clinical or epidemiological behavior.

In recent years, there has been a shift in the classic patterns of RSV circulation, with atypical outbreaks and earlier seasonal onset in several countries following the COVID-19 pandemic [6], [7]. In Latin America, this phenomenon has been documented in the Southern Cone, where countries such as Chile, Argentina, and Uruguay have reported an earlier and more aggressive increase in cases than in previous seasons [8]. Chile is of particular interest due to its consolidated surveillance system, access to public data, and a public health structure that combines mass communication strategies with non-pharmaceutical measures.

Chile became the first country in the Americas to officially incorporate passive immunization with nirsevimab in newborns and infants into the public health system as part of its national RSV control strategy. This long-acting monoclonal antibody is effective in preventing severe illness in infants during their first seasonal exposure to the virus [9], [10]. However, the winter of 2025 in Santiago revealed its limitations: A significant epidemic outbreak was recorded in the one- to four-year-old population, not covered by the strategy, with a positivity rate of 20% in epidemiological week 25 and with RSV being the predominant respiratory agent, according to 1618 samples processed between June 16 and 22 [11].

International evidence supports the use of universal immunization with nirsevimab as a cost-effective measure that can significantly reduce hospitalizations and intensive care admissions [12]–[17]. These findings reinforce the relevance of the Chilean strategy and the need for local economic evaluations to optimize its sustainability, thereby expanding coverage and enhancing preventive planning.

Mathematical models are essential tools for understanding and predicting the transmission dynamics of infectious diseases. Their selection depends on the availability and quality of data, as well as the biological and epidemiological characteristics of the pathogen under study [1], [18]. In the case of RSV, compartmental SEIRS-type models (susceptible–exposed–infected–recovered) have been widely used, allowing the incorporation of specific parameters to suit the analytical objective. When abrupt changes in dynamics occur, induced by external factors such as prevention campaigns or seasonal variations, the use of impulsive differential equations provides a more realistic representation by integrating discrete events that suddenly modify transmission or recovery rates. Validation and calibration of these models with real epidemiological data enhance the accuracy of projections and support the design of control strategies tailored to different contexts [19]–[21].

There is a wide variety of mathematical models that simulate the dynamics of RSV, including SEIR-type compartmental models, models based on classical ordinary differential equations [22]–[24], and fractional differential equations with optimal control [25], [26], among others. The model presented in this proposal employs impulsive differential equations and incorporates hospital capacity as one of its compartments, distinguishing it from existing models.

The objective of this study is to analyze, using a mathematical model based on SEIRS-type impulsive differential equations, the combined impact of pharmacological interventions, such as passive immunization with nirsevimab, and non-pharmacological interventions, such as prevention campaigns aimed at modifying population behavior, on the transmission dynamics of RSV in the Chilean child population, incorporating the interaction between epidemiological and behavioral variables and hospital capacity.

## 2. Materials and method

An important aspect to consider in modeling with strategic (generalist) infectious disease models based on the SEIRS compartmental model is that, in general, these models assume constant transmission and recovery rates. In this case, for each outbreak (new year), the curve of new cases per unit of time, or prevalence, shows the traditional bell-shaped pattern, where a higher transmission rate (*β*) implies an earlier and higher peak. Thus, for example, the graphs in Figure 1 (with data extracted from Chile) for 2019, 2022, and 2023 could be explained by an increasing *β*, resulting from the combination of decreasing mitigation measures and seasonal and population behavior.

**Figure 1.**
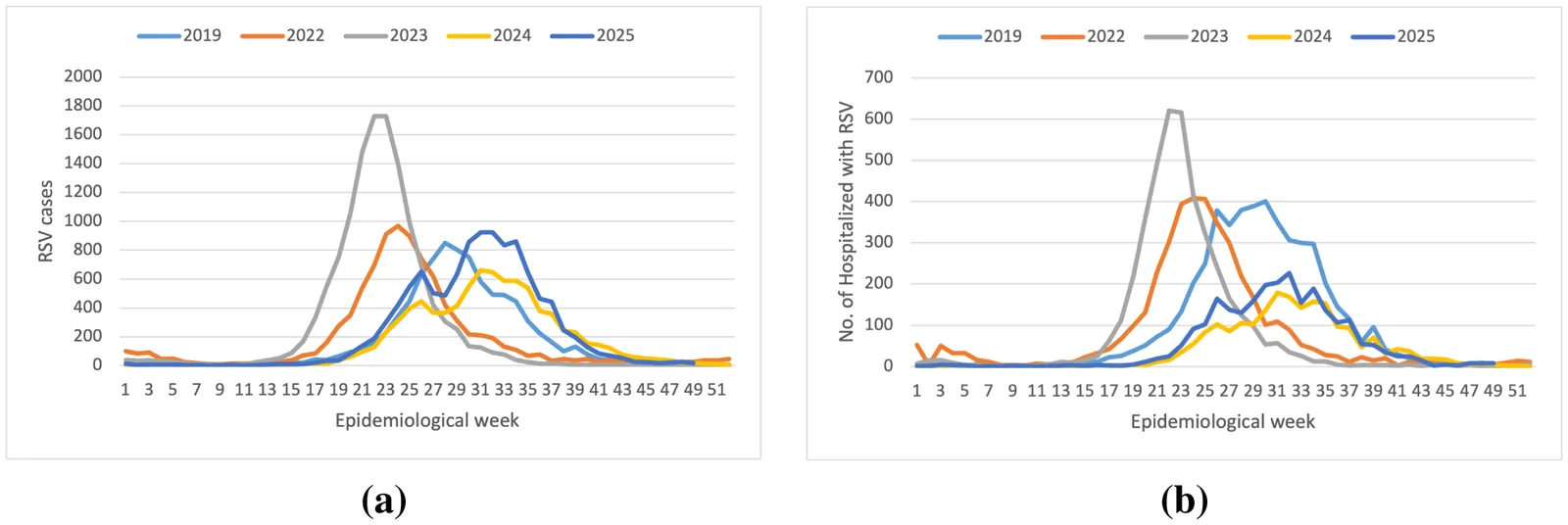
Graphs created from data reported by MINSAL [11].

Thus, the general dynamics of the proposed model, which represents RSV, are expressed by the susceptible (*S*), exposed (*E*), infectious (*I*), and recovered (*R*) states, the latter with temporary immunity. The initial (fixed) transmission rate is β_*_, where we propose a dynamic rate [27]–[32] expressed by $\beta^{p,q}=\beta_{*}^{q}(P_{*}/P)$ where *P* is the behavior influenced by individuals' risk perception regarding the RSV, P^*^ is the quantified average population's behavior, and the superscript *q* is the epidemiological three-month period of the year, with *q* ∈ {1, 2, 3, 4}. The functional form of the transmission rate is based on a simplification of the works by [33], [34], which has been applied in various epidemiological contexts in the work by [27],[35]–[37]. The average time that an individual remains in the exposed and infectious states is 1/*δ* and 1/*γ*, respectively. Parameter 1/*ψ* is the average time that an individual remains immune before becoming susceptible to the disease again (see first row of Figure 2). In addition, hospitalization (*H*), whose rate is given by *σ^q^*, and hospital collapse (*H_C_*), which represents the number of people who need hospitalization and cannot access it, are incorporated. Hospital collapse is triggered by the function Γ(*H*) defined by [*H*/H_*_]|*σ^q^* − *γ_h_*|, where [·] is integer part function and H* is the hospital bed capacity. The proposed function (Γ(*H*)) is designed to “activate” when the population size of hospitalized patients *H* exceeds hospital capacity H^*^, thereby initiating hospital collapse; this is why we have used the integer part function, and once it is activated, it will depend primarily on the difference between the flows that increase or decrease hospital collapse at three of the *σ*−*γ_h_* rates. The entry (births) and exit rates of the risk group are given by Λ and *µ*, respectively. The mortality rate is given by *d* (natural deaths not attributable to RSV disease, as provided in all compartments) (see Figure 2).

**Figure 2.**
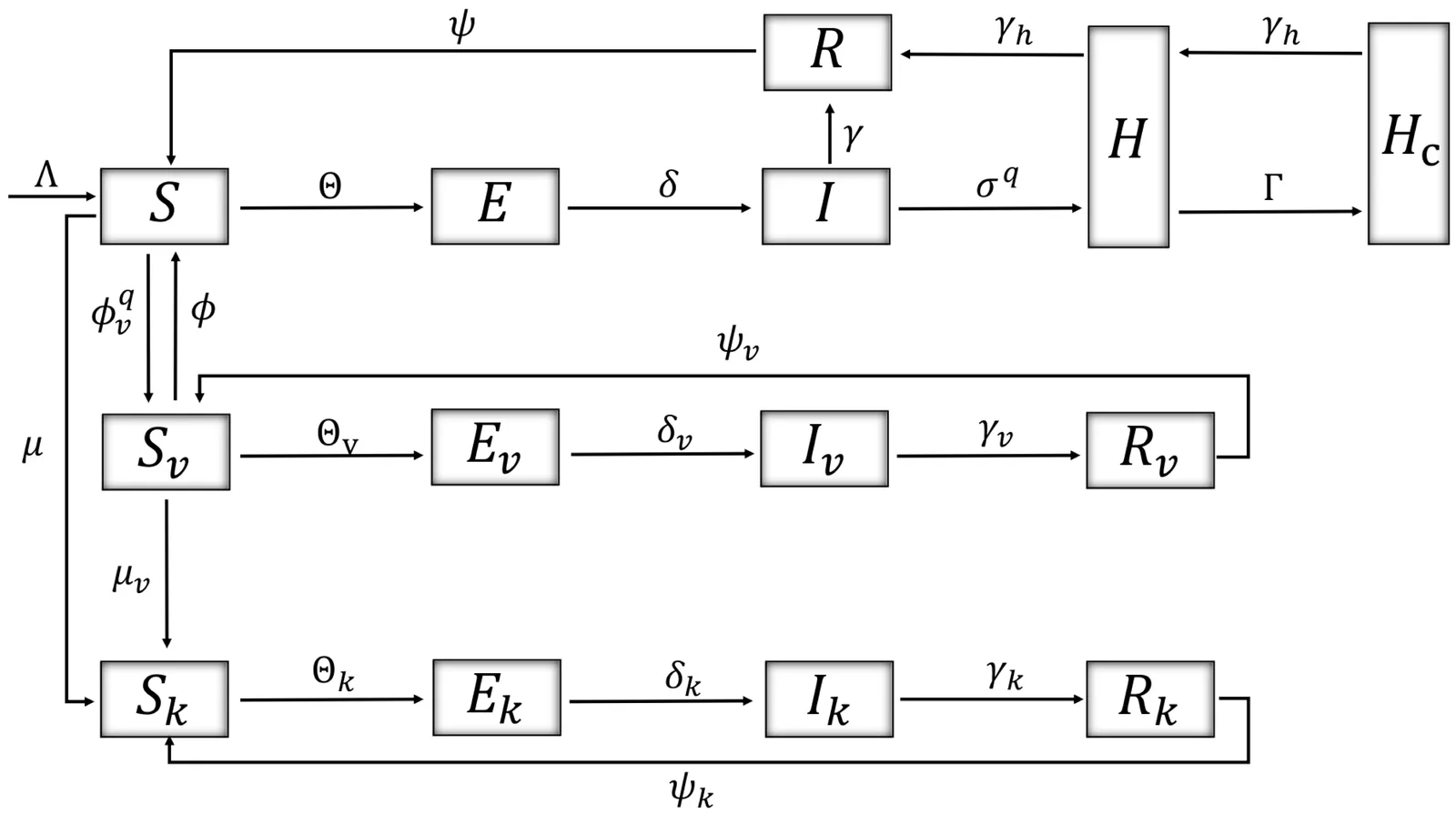
Flowchart, where $\Theta =\beta^{p,q}SI_{k}/N$, $\Theta_{v}=\beta_{v}^{p,q}S_{v}I_{k}/N$, $\Theta_{k}=\beta_{k}^{p,q}S_{k}(I+I_{v}+I_{k})/N$, and Γ=[*H*/H_*_]|*σ^q^* − *γ_h_*|, where [·] is integer part function.

Individuals (risk group) who receive the monoclonal antibody to combat RSV are denoted by the subscript *v* for any compartment (see second row of Figure 2). For simplicity of the model, we have assumed that this method is applied to susceptible individuals at a rate of $\varphi_{\nu }^{q}$, and the average time of effectiveness is given by 1/*φ*. We have considered that there is the possibility of becoming infected with RSV even when immunized at a variable transmission rate expressed by $\beta_{v}^{p,q}=\beta_{*,v}^{q}(P_{*}/P)$, where $\beta_{*,v}$ is the initial (fixed) transmission rate with immunization. Regarding the average times of exposure, infectivity, and immunity after infection, they are given by 1/*δ_v_*, 1/*γ_v_*, and 1/*ψ_v_*, respectively. It should be noted that by being immunized with this antibody, we assume that individuals infected with RSV are not referred for hospitalization. This assumption stems from the fact that if there are cases, they are typically cases of high-risk infants. Therefore, for the sake of simplicity of the model, we do not consider those cases. Among those vaccinated, the rate of exit from the risk group is denoted by *µ_v_*.

The non-risk group is denoted by the subscript *k*. This group is fed by people who leave the risk group through rates *µ* and *µ_v_*, from the unvaccinated and vaccinated, respectively. The transmission rate is given by $\beta_{k}^{p,q}=\beta_{*,k}^{q}(P_{*}/P)$, where $\beta_{*,k}$ is the initial (fixed) transmission rate. Finally, 1/*δ_k_*, 1/*γ_k_*, and 1/*ψ_k_* denote the average times for exposure, infectiousness, and removal, respectively (see third row of Figure 2).

The dynamics of the disease are continuous in time, while the awareness campaigns (which improve behavior *P* in a factor *α*) are discrete in time, i.e., every few instants. Therefore, the model is expressed by a system of impulsive differential equations; see (2.1).

$$\left\{ \begin{array}{l} \begin{array}{l} \dot{S}(t)=\Lambda -\beta^{p,q}SI_{k}/N-(µ+\varphi_{v}^{q}+d)S+\psi R+\varphi S_{v}\\ \dot{E}(t)=\beta^{p,q}SI_{k}/N-(\delta +d)E\\ \dot{I}(t)=\delta E-(\gamma +\sigma^{q}+d)I\\ \dot{H}(t)=\sigma^{q}I-(\Gamma +\gamma_{h}+d)H+\gamma_{h}H_{c}\\ {\dot{H}}_{c}(t)=\Gamma H-(\gamma_{h}+d)H_{c}\\ \dot{R}(t)=\gamma I+\gamma_{h}H-(\psi +d)R\\ {\dot{S}}_{v}(t)=\varphi_{v}^{q}S-\beta_{v}^{p,q}S_{v}I_{k}/N-({µ}_{v}+\varphi +d)S_{v}+\psi_{v}R_{v}\\ {\dot{E}}_{v}(t)=\beta_{v}^{p,q}S_{v}I_{k}/N-(\delta_{v}+d)E_{v}\\ {\dot{I}}_{v}(t)=\delta_{v}E_{v}-(\gamma_{v}+d)I_{v}\\ {\dot{R}}_{v}(t)=\gamma_{v}I_{v}-(\psi_{v}+d)R_{v}\\ {\dot{S}}_{k}(t)=µS+{µ}_{v}S_{v}-\beta_{k}^{p,q}S_{k}(I+I_{v}+I_{k})/N-dS_{k}+\psi_{k}R_{k}\\ {\dot{E}}_{k}(t)=\beta_{k}^{p,q}S_{k}(I+I_{v}+I_{k})/N-(\delta_{k}+d)E_{k}\\ {\dot{I}}_{k}(t)=\delta_{k}E_{k}-(\gamma_{k}+d)I_{k}\\ {\dot{R}}_{k}(t)=\gamma_{k}I_{k}-(\psi_{k}+d)R_{k}\\ \dot{P}(t)=-\lambda_{1}(P-P_{*})+\lambda_{2}(I+I_{v}+I_{k}+H+H_{c})/N \end{array}\}\;t\notin \{t_{n}\}\\ P(t^{+})=(1+\alpha )P\;\}\;t\in \{t_{n}\}. \end{array}\right.$$ (2.1)

where {*t_n_*} represents the finite set of intervention times for preventive campaigns. Note that the equation governing the dynamics of perception *P*(*t*) in the first part of (1) accounts for two key mechanisms: a natural prevention term (unrelated to educational interventions), proportional to the total infectious population (with constant *λ*_2_), which may reflect fear-driven behavior; and a subtractive term representing a gradual return to an intrinsic cultural-environmental baseline value P_*_ with a rate *λ*_1_. This structure reveals how the design of awareness campaigns draws inspiration from pharmacokinetic principles. The summary of model states and parameters is summarized in Tables 1 and 2, respectively. The data presented in Table 2 are supported by the cited scientific sources, which establish a range of possibilities; consequently, a value close to the mean is generally taken as the baseline. The data used, as detailed in Table 2, are divided into three categories: epidemiological data, hospitalization data, and human behavior data. The epidemiological data were primarily drawn from studies and information sources specific to Chile, supplemented by global literature. The hospitalization data came exclusively from Chilean sources, while the data on human behavior were drawn from existing literature spanning various contexts.

**Table 1.** Variables of the model.

	**Susceptible**	**Exposed**	**Infectious**	**Hospitalized**	**Hosp. collapse**	**Removed**
**No Vaccine**	*S*	*E*	*I*	*H*	*H_c_*	*R*
**Vaccinated**	*S_v_*	*E_v_*	*I_v_*	–	–	*R_v_*
**Risk-free**	*S_k_*	*E_k_*	*I_k_*	–	–	*R_k_*

**Table 2.** Model parameters (**P**). In the last column, **ref**_1_ indicates references [22], [38]–[40] and **ref**_2_ the references [27], [35], [41]. In the last row, *U* denotes a dimensionless quantity. We use *d* for days. AC stands for author chosen.

**P**	**Description / Unit**	**Range**	**Value**	**ref.**
*Epidemiological parameters*				
Λ	Risk group entry rate (birth rate) / *d*^−1^	1/(78*52*7)*[0,4]	1/(78*52*7)	moore2014modelling
*d*	Death rate not attributable to RSV disease / *d*^−1^	1/(78*52*7)*[0,4]	1/(2*52*7)	AC
1/*µ_x_*	Average time in risk group	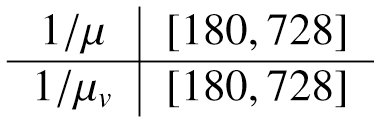	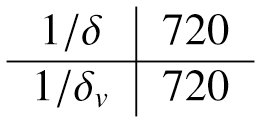	
	(with vacc., *x* = *v*) / *d*			[22]
$\varphi_{v}^{q}$	Vaccination (vacc.) rate / *d*^−1^	(1/120) · [0, 0.25]	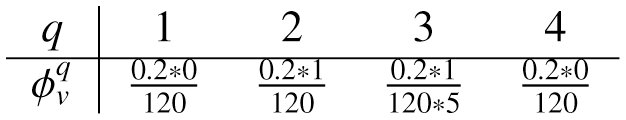	[42]
1/*φ*	Average immunization time after			
	vacc. / *d*	[120,180]	180	[43]
$\beta_{*,x}^{p,q}$	Intrinsic transmission rate without vacc.			
	(with vacc. (*x* = *v*), non-risk group (*x* = *k*)) /*d*^−1^	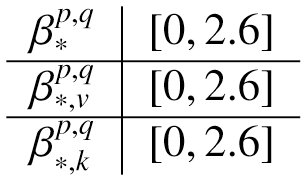	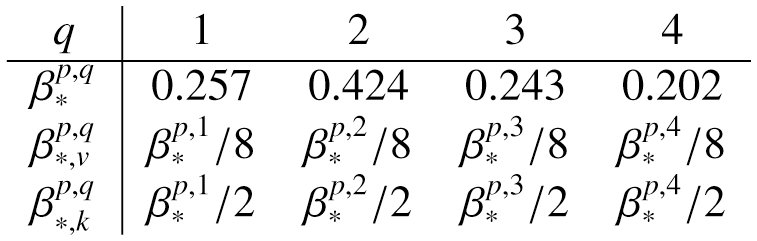	**ref**_1_
1/*δ_x_*	Average latency time without minting			
	(with vacc. (*x* = *v*), non-risk group (*x* = *k*)) / *d*	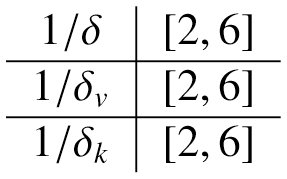	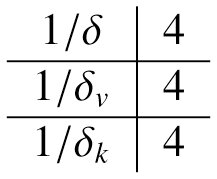	**ref**_1_
1/*γ_x_*	Average time an individual remains infectious without vacc.			
	(with vacc. (*x* = *v*), non-risk group (*x* = *k*)) / *d*	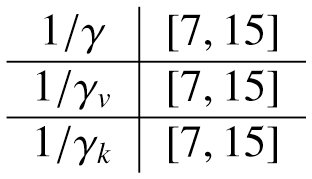	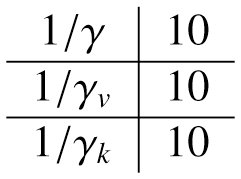	**ref**_1_
1/*ψ_x_*	Average duration of natural immunity without vacc.			
	(with vacc. (*x* = *v*), non-risk group (*x* = *k*)) / *d*	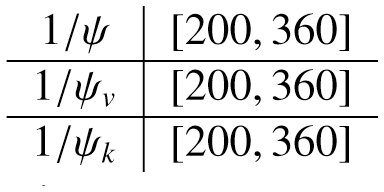	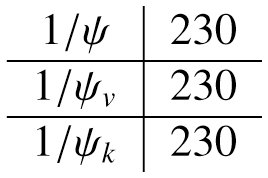	**ref**_1_
*Hospitalization parameters*				
1/*γ_h_*	Average length of time an individual			
	remains hospitalized / *d*	[7,15]	8	[44]
*σ^q^*	RSV hospitalization rate / *d*^−1^	[0,1]	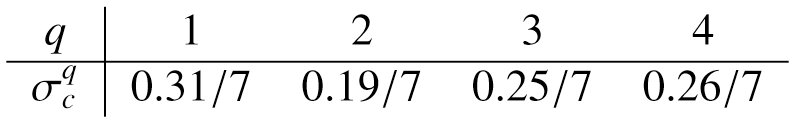	[45]
*Behavioral parameters*				
*λ*_1_	Rate of resistance to change / *d*^−1^	[0,1]	0.03	**ref**_2_
*λ*_2_	Reaction rate / *d*^−1^	[0,1]	0.03	**ref**_2_
P_*_	Natural individual behavior / *P*		0.5	**ref**_2_
*α*	Behavioral improvement toward RSV			
	disease / *U*	[0,1]	0, 0.03 and 0.1	AC
*t_n_*	Time instants where prevention campaigns occur. / *d*		7*52*k, *k* ∈ ℕ_0_	AC

## 3. Results

The numerical results of the model are presented. Time is given by (epidemiological) weeks, and the population has been normalized (*N* = 1) and constant (Λ = *d*), focusing on the risk group zero to two years of life. The initial conditions used for this section were *S*(0) = 0.01499, *I*(0) = 0.00001, *S_k_*(0) = 0.985, *P*(0) = P_*_, and all others were zero (*E*(0) = *H*(0) = *H_c_*(0) = *R*(0) = *S_v_*(0) = *E_v_*(0) = *I_v_*(0) = *R_v_*(0) = *E_k_*(0) = *I_k_*(0) = *R_k_*(0) = 0).

Figure 3 illustrates the dynamics of RSV disease following the implementation of a vaccination system, excluding prevention campaigns aimed at modifying behavior toward the disease. The total number of RSV cases is shown in light blue, i.e., the sum of infectious (*I* and *I_v_*, red line), hospitalized (*H*, blue line), and persons in hospital collapse (*H_c_*, black line). Figure 3(a) illustrates the base case, i.e., without vaccination or prevention campaigns, where an epidemiological curve is observed annually, varying the number and the instant at which the peak of contagion occurs. The three curves show a hospital collapse (see the black curves), with the highest value corresponding to the amplitude and peak of the infectious curve. Figures 3b–d incorporate a vaccination system applied in the second and third quarters of the epidemiological year, i.e., when the disease has the highest number of active cases. After using a vaccination rate of 0.2/120 during the second quarter and one-fifth (0.2/(120*5)) during the third quarter (see Figure 3b), a decrease in cases is observed in all four years. However, from the second year onward, hospitals become overwhelmed again, though to a lesser extent than in the base case, which can be attributed to a “relaxation” in the population. After an increase in the vaccination rate in the second and third quarters (a fivefold increase in each quarter) to reach a higher percentage of the target population, a significant decrease in the number of cases is observed (see Figure 3c), leading to a substantial reduction in hospital collapse (“disappears”) from the first year after vaccination. However, in the fourth year, it reappears at a lower level than in the base case. Finally, in Figure 3d, after doubling the vaccination rate compared to case (c), it can be seen that, given even greater coverage of people in the risk group (which is ideal), hospital collapse disappears from the first year of implementation of the vaccination system and does not reappear.

**Figure 3.**
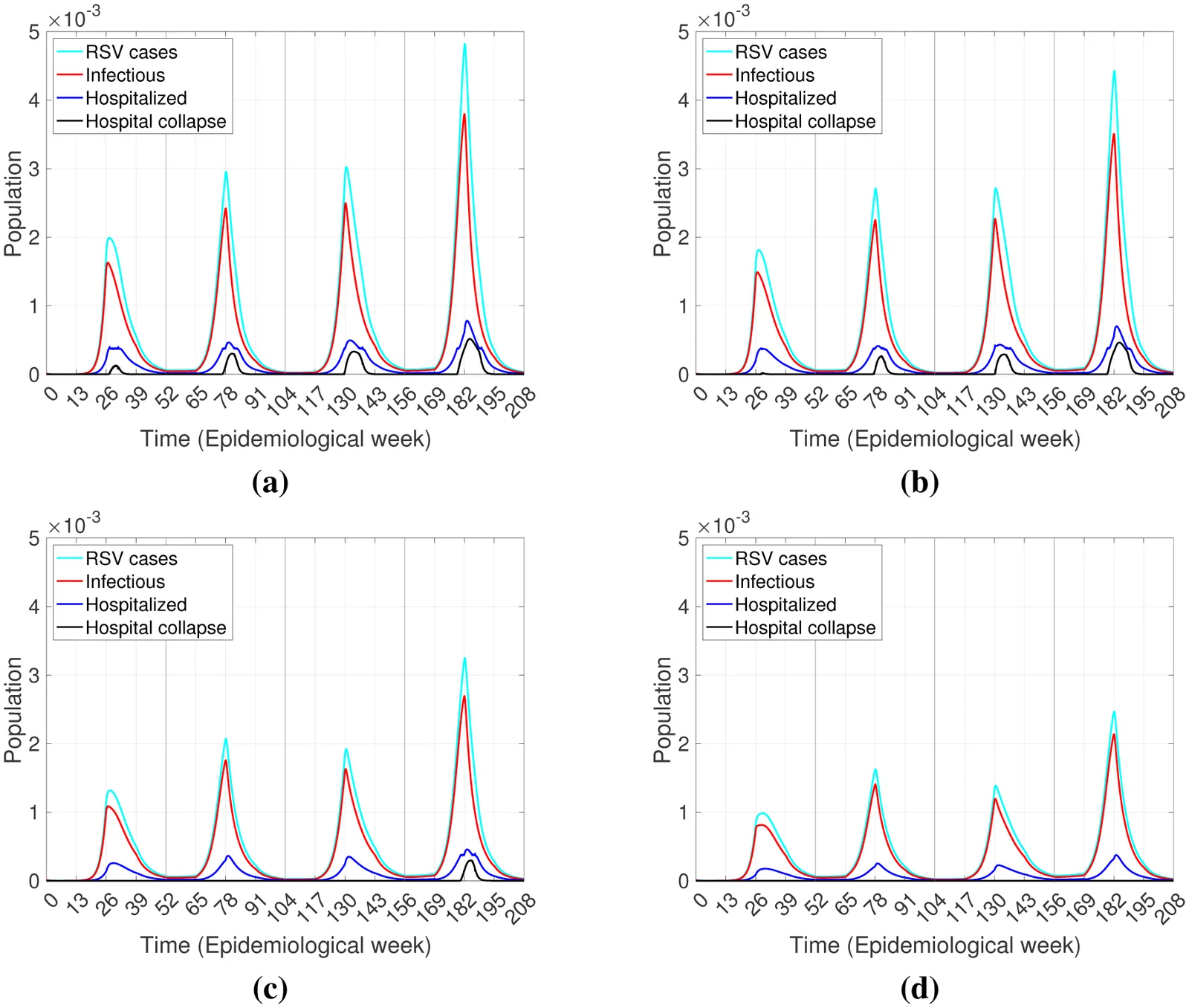
RSV cases after vaccination. Figure (a) without vaccination, (b) vaccination with rates given in Table 2, (c) vaccination rate increased five times, (d) vaccination rate increased 10 times.

Figure 4 illustrates the impact of people's behavior on the disease, excluding vaccination. Figure 4a shows the base case. In cases where non-pharmaceutical prevention campaigns are implemented (Figures 4b–d), a 3% increase (*α* = 0.03) in the quantified improvement in behavior has an impact on the outbreak in the first and third years, smoothing and decreasing the epidemiological curve (even eliminating hospital queues). However, in the second and fourth years there is a reduction, although not significant (see Figure 4b). In cases where campaigns are developed that positively impact people's behavior (resulting in a 10% increase (*α* = 0.1)), there is a significant decrease in cases, also in the first and third years. Still, during the second and fourth years, the reduction remains insignificant (see Figure 4c). For case (c), if we assume that the reaction speed also improves from 0.03 to 0.09, we obtain a case similar to the previous one, with a reduction in the peaks in the curves (see Figure 4d). The dynamics of people's behavior (*P*) are shown in Figure 4e. It can be seen that, although prevention campaigns are carried out every year, people tend to return to their natural behavior. This effect has implications for the transmission rate, which varies with behavior (see Figure 4f).

**Figure 4.**
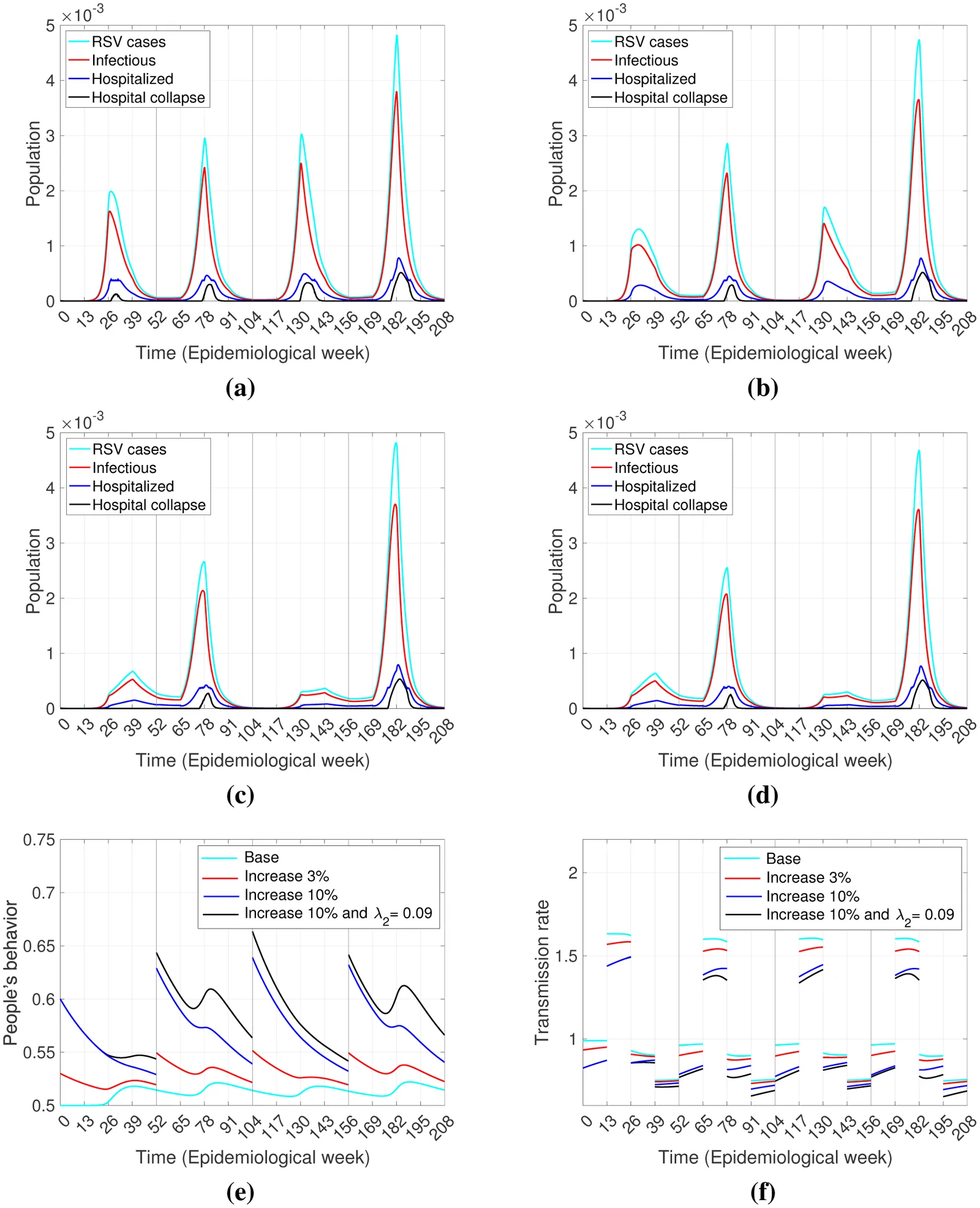
RSV cases are changing people's behavior towards the disease (without considering vaccination). Figure (a) base case, (b) increase in behavior (quantified) by 3%, (c) increase in behavior (quantified) by 10%, (d) increase in behavior (quantified) by 10% and an increase in speed of reacting (*λ*_2_) from 0.03 to 0.09, (e) the curve of people's behavior (*P*(*t*)) based on cases (a)–(d), and (f) the transmission rate dependent on people's behavior based on cases (a)–(d).

Figure 5 illustrates the impact of integrating vaccination campaigns with non-pharmaceutical prevention measures that positively influence people's behavior. The base case is shown in Figure 5a. In Figures 5b–d correspond to Figures 3b–d respectively, after considering an increase in behavior (quantified) by 10% (*α* = 0.1) and an increase in the speed of reacting (*λ*_2_) from 0.03 to 0.09. Observe that the decrease in positive cases is significantly higher and that the hospital collapse in case (c) is practically null. In particular, after achieving broad vaccination coverage and non-pharmaceutical prevention campaigns that positively impact the population (see Figure 5d), the disease is controlled (with small outbreaks), with few hospital cases, leading to no hospital collapse.

**Figure 5.**
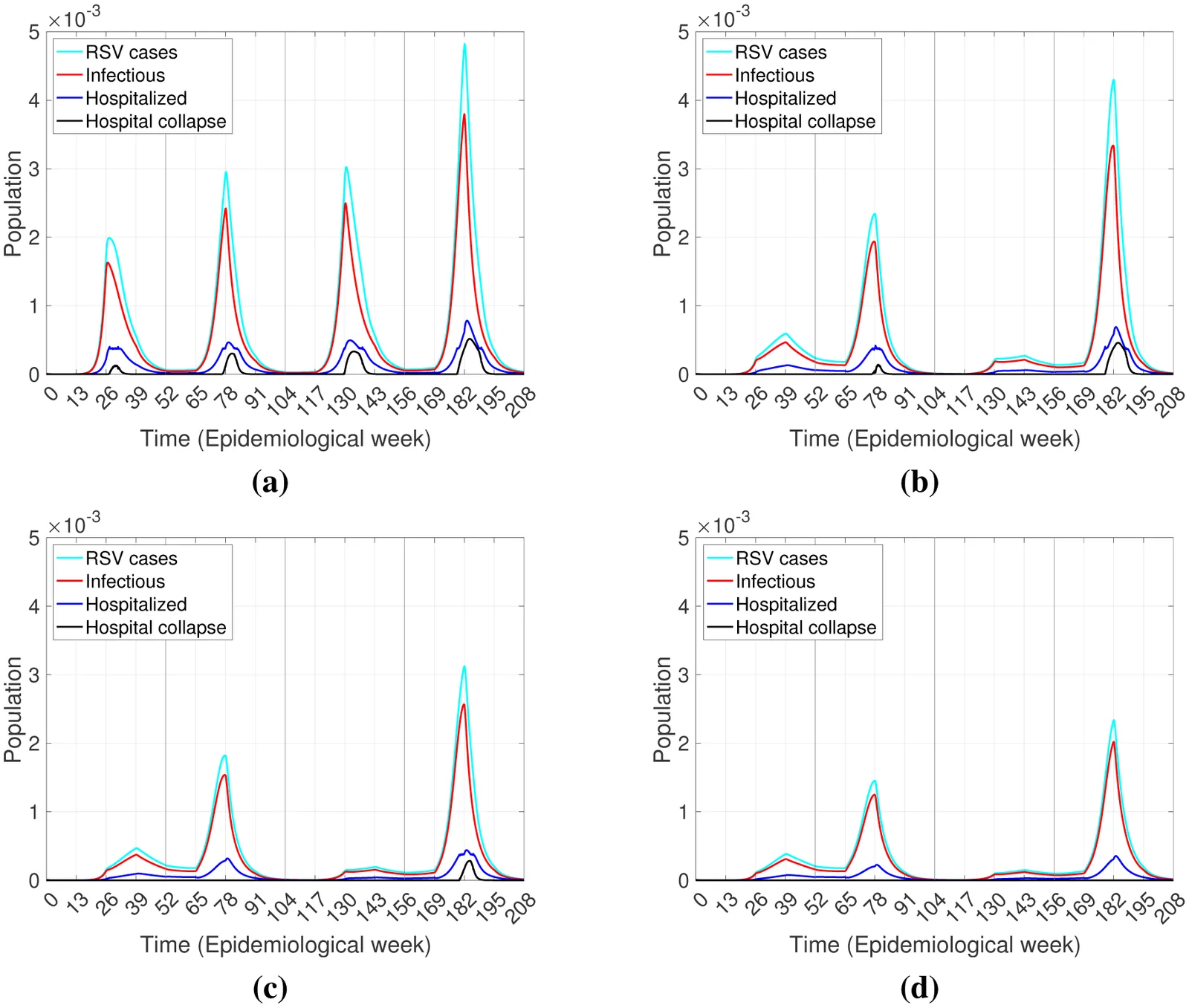
RSV cases after the application of vaccination and changes in people's behavior towards the disease. a) base case, (b) vaccination with rates given in Table 2, an increase in behavior (quantified) by 10% and an increase in the speed of reacting (*λ*_2_) from 0.03 to 0.09. (c) vaccination rate increased by five times, an increase in behavior (quantified) by 10% and an increase in the speed of reacting (*λ*_2_) from 0.03 to 0.09, and (d) vaccination rate increased by 10 times, an increase in behavior (quantified) by 10% and an increase in the speed of reacting (*λ*_2_) from 0.03 to 0.09.

The results presented in Figures 3–5 show that the strategies incorporated into the impulsive model indicate that risk-perception measures have a lesser impact than pharmaceutical measures. However, these strategies serve as an essential complement to ensure that pharmaceutical measures have a greater impact on reducing hospital admissions. A comparison of simulated and observed cases is evident when Figure 1, which presents cases in Chile from 2019 to 2025, is contrasted with the simulation results shown in Figures 3–5.

### 3.1. Sensitivity analysis

A local sensitivity analysis was conducted to examine the influence of various parameters on hospital burden. The parameters analyzed were those that vary quarterly during the epidemiological year (those marked with the superscript *q*) and those associated with human behavior (see Figure 6).

**Figure 6.**
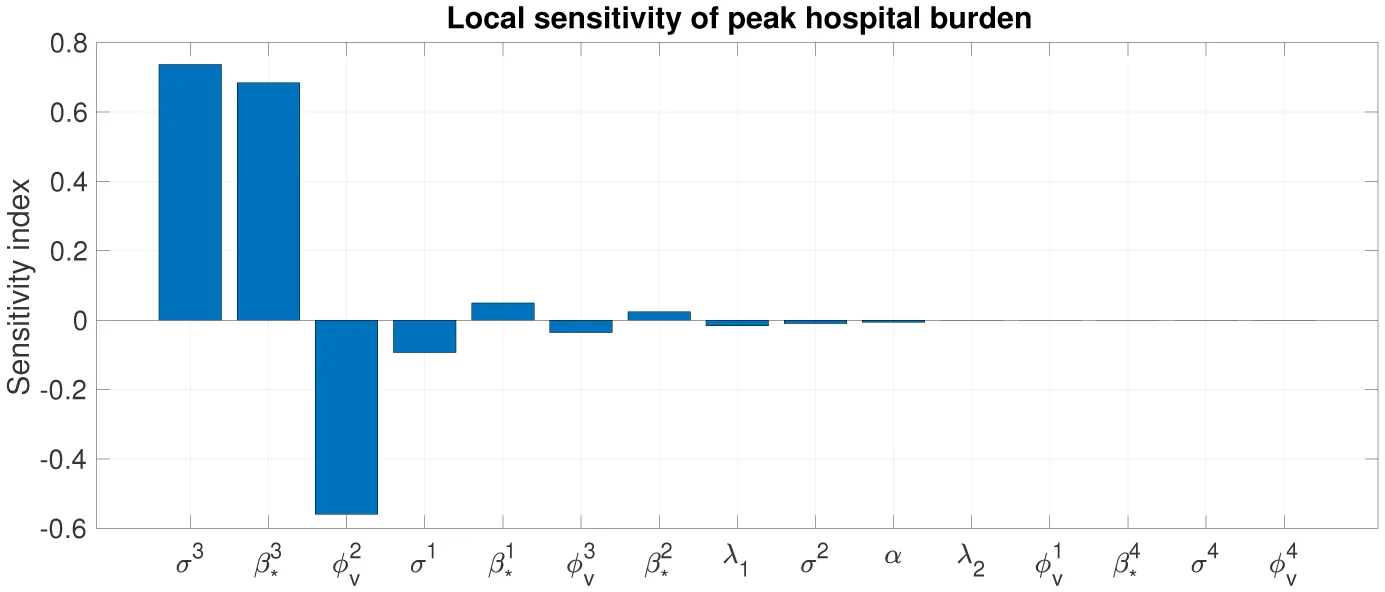
Local sensitivity analysis.

Figure 6 shows that the parameters with the greatest influence on the peak number of RSV-associated hospitalizations are *σ^q^* and $\beta_{*}^{q}$, particularly those corresponding to the epidemiological period in the third quarter of the year. If these parameters increase by 1% during that period, the peak number of hospitalizations rises by 74% and 69%, respectively.

The greatest protective effect against the peak in hospitalizations is demonstrated by the parameter *φ_v_*, corresponding to vaccination during the second quarter, which highlights the importance of vaccinating before the peak in infections occurs (which occurred in the third quarter). Furthermore, among the preventive measures, this parameter in the model appears to be the most effective.

The remaining parameters have sensitivities close to zero, so their influence on the peak in hospital admissions is not significant under the conditions considered. In particular, since the parameters related to human behavior also have low sensitivities, it can be concluded that pharmaceutical strategies have a greater influence than non-pharmaceutical strategies on the peak in hospital admissions.

## 4. Discussion and conclusion

Human RSV remains a major cause of acute respiratory infections in young children worldwide, particularly in low- and middle-income countries, where it is responsible for a high proportion of hospitalizations due to bronchiolitis and pneumonia [1], [2]. Its endemic nature and predictable seasonal circulation patterns have been disrupted in recent years, with atypical outbreaks and shifts in peak incidence reported following the COVID-19 pandemic [6], [7].

The modeling results presented here indicate that in the absence of preventive measures, pharmaceutical or non-pharmaceutical, RSV transmission can escalate to levels capable of overwhelming hospital capacity, as reflected in the base scenario (Figure 3a). Hospital overcrowding is context-dependent, varying with the number of available beds, demographic structure, and healthcare accessibility. In our simulations, the introduction of pharmaceutical prevention, represented by immunization with monoclonal antibodies such as nirsevimab, reduced both incidence and hospitalization burden, with hospital collapse disappearing once high coverage levels were achieved [9], [10], [12]. These findings align with economic evaluations indicating that universal nirsevimab administration is cost-effective and can substantially reduce severe outcomes [13], [17].

Non-pharmaceutical interventions, modeled here as behavioral modifications induced by public health campaigns, also demonstrated a meaningful reduction in transmission and hospitalization rates (Figure 4). This is consistent with evidence showing that community-level preventive behaviors, such as reducing physical contact during peak circulation, can influence epidemic dynamics [8]. However, our results indicate that such measures alone are insufficient to eliminate the risk of hospital collapse, underscoring the need for integrated approaches.

When both pharmaceutical and non-pharmaceutical strategies were applied jointly, the model projected the elimination of hospital collapse and a marked reduction in epidemic amplitude (Figure 5). This synergistic effect is consistent with observations from Chile. After a significant RSV associated hospital burden in 2023, the Ministry of Health implemented a national immunization program with nirsevimab for infants under six months of age in 2024, complemented by public health communication measures. Data from the Metropolitan Surveillance Network showed reductions in RSV positivity and hospitalizations among high-risk groups during the subsequent winter season [11].

A key strength of this work is the incorporation of impulsive differential equations to capture the discrete nature of behavioral interventions alongside the continuous dynamics of viral transmission. This hybrid approach allowed us to model both the temporal targeting of prevention campaigns and the seasonally variable transmission rate, integrating population behavior as an explicit dynamic variable. Moreover, by including hospitalization and hospital collapse as model compartments, the framework connects epidemiological projections directly with healthcare system impact, enhancing its relevance for policy planning.

The model focuses on a high-risk pediatric subgroup (zero to two years old) whose primary source of infection is through the non-risk group (over two years old). This was considered because empirical evidence indicates that older age groups contribute substantially to RSV transmission. For example, [46] found that household transmission often originates from parents or older siblings. In addition, epidemiological surveillance in Chile during the 2025 RSV season documented a high caseload among children aged one to four years, with a 20% RSV positivity rate reported in epidemiological week 25 [11].

Within the limitations of the model, we note that hospital capacity thresholds were modeled as fixed values, without accounting for seasonal variability in healthcare resources or the system's possible adaptive responses. It was assumed that vaccine efficacy and behavior change parameters were homogeneous across the population, failing to capture heterogeneity in social determinants, geographic distribution, or differential access to interventions. In addition, the simulations used Chilean epidemiological patterns as a reference, which could limit direct generalization to countries with different climatic, demographic, or health profiles.

Another simplifying assumption in the model is that infants receiving nirsevimab are fully protected against RSV-related hospitalizations during their first season. While clinical trials have reported high efficacy in preventing medically attended RSV infections, including a 62% reduction in RSV-related hospitalizations reported in the MELODY trial [47], breakthrough hospitalizations can still occur, particularly in high-risk infants. Therefore, this assumption may lead to a slight overestimation of the intervention's impact on hospital demand. Future work could incorporate partial protection estimates, depending on real-world effectiveness data from the Chilean immunization program as they become available.

Despite these limitations, the model offers a flexible framework for evaluating RSV control strategies in diverse contexts. The results reinforce the necessity of integrated prevention approaches—combining pharmaceutical interventions such as nirsevimab with sustained public health campaigns—to achieve long-term control of RSV transmission and avoid seasonal hospital crises [8], [12], [13], [15]. For policymakers, this means prioritizing high coverage in target populations while maintaining year-round communication strategies to sustain protective behaviors [6], [48].

For future research, the model could be extended to account for waning immunity in older cohorts, co-circulation with other respiratory viruses, and real-time calibration using sentinel surveillance data [1], [18]. Such enhancements would further improve predictive accuracy and support adaptive intervention strategies in response to shifts in RSV epidemiology, such as those observed post-COVID-19 [7].

In conclusion, controlling RSV requires a sustained and coordinated public health strategy that combines pharmaceutical interventions, such as immunization, with non-pharmaceutical measures that promote and maintain protective behaviors within the community. This combined application reduces transmission, limits the severity of outbreaks, and helps maintain the functionality of healthcare systems during seasonal peaks. The results demonstrate that relying on a single intervention is insufficient; long-term control depends on the consistent, simultaneous application of both strategies.

## Use of AI tools declaration

The authors declare they have not used Artificial Intelligence (AI) tools in the creation of this article.
